# Post-exercise Recovery Methods Focus on Young Soccer Players: A Systematic Review

**DOI:** 10.3389/fphys.2021.505149

**Published:** 2021-05-20

**Authors:** Julio Calleja-González, Juan Mielgo-Ayuso, Álvaro Miguel-Ortega, Diego Marqués-Jiménez, Miguel Del Valle, Sergej M. Ostojic, Jaime Sampaio, Nicolás Terrados, Ignacio Refoyo

**Affiliations:** ^1^Department of Physical Education and Sports, Faculty of Education and Sport, University of the Basque Country (UPV/EHU), Vitoria-Gasteiz, Spain; ^2^Department of Health Science, Faculty of Health Sciences, University of Burgos, Burgos, Spain; ^3^Faculty of Education, University of the Middle Atlantic, Las Palmas, Spain; ^4^Academy Department, Deportivo Alavés SAD, Vitoria-Gasteiz, Spain; ^5^Department of Health Sciences, Faculty of Health Sciences, Universitat Oberta de Catalunya, Barcelona, Spain; ^6^Department of Cellular Morphology and Biology, Universidad de Oviedo, Oviedo, Spain; ^7^Center for Health, Exercise and Sport Sciences, Belgrade, Serbia; ^8^Research Center in Sports Sciences, Health Sciences and Human Development (CIDESD), University of Trás-os-Montes e Alto Douro, Vila Real, Portugal; ^9^Regional Unit of Sports Medicine, Aviles and Health Research Institute of the Principality of Asturias (ISPA), Aviles, Spain; ^10^Department of Sports, Faculty of Physical Activity and Sports Sciences (INEF), Polytechnic University of Madrid, Madrid, Spain

**Keywords:** regeneration, youth, athletes, recovery, fatigue, soccer

## Abstract

**Background:** Prescription of post-match or post-training recovery strategies in young soccer players is a key point to optimize soccer performance. Considering that the effectiveness of recovery strategies may present interindividual variability, scientific evidence-based recovery methods and protocols used in adults are possibly not applicable to young soccer players. Therefore, the current systematic review primarily aimed to present a critical appraisal and summary of the original research articles that have evaluated the effectiveness of recovery strategies in young male soccer players and to provide sufficient knowledge regarding the effectiveness of the recovery methods and strategies.

**Methodology:** A structured search was carried out following the Preferred Reporting Items for Systematic Review and Meta-Analyses (PRISMA) guidelines until November 31, 2020, using the next data bases: WOS, PubMed, Cochrane Library, Evidence Database (PEDro), Evidence Based Medicine (EBM) Search review, EMBASE, and Scopus. There were no filters applied.

**Results:** A total of 638 articles were obtained in the initial search. After the inclusion and exclusion criteria, the final sample was 10 articles focusing on recovery in young male players.

**Conclusions:** Neuromuscular performance can be recovered using WVB but not with SS, and water immersion protocols may also be useful, but their positive effects are not significant, and it is unable to distinguish the best water immersion method; match running performance maintenance may be achieved using water immersion protocols but no other recovery methods have been investigated; EIMD and inflammatory responses could be positively affected when water immersion and AR are applied, although SS seems to be ineffective; perceptual responses also seem to be better with CWI and WVB, but contradictory results have been found when AR is applied, and SS had no positive impact. Finally, it is important to consider that AR strategies may modify HR response and soccer-specific performance.

## Introduction

A soccer competitive match induces greater magnitudes of exercise-induced muscle damage (EIMD), delayed-onset muscle soreness (DOMS), biochemical changes, and neuromuscular alterations (Silva et al., 2018). Moreover, congested fixture schedules in elite soccer results in residual fatigue and underperformance in ensuing competition due to insufficient recovery time (Carling et al., 2015), so it is crucial to have clear insight into players' recovery during this short-term match congestion periods (Nédélec et al., 2012; Kellmann et al., 2018), and to determine the balance between training and recovery to achieve the athletes' desired adaptations (Stanley et al., 2013).

Considering that the recovery status in professional soccer players is influenced by match running activity, the physical characteristics of each player, the training workload between matches +24 and +72 h, and/or the potential use of post-competition recovery strategies (Carling et al., 2018), three main efforts must be initiated to improve recovery. On the one hand, coaches must adjust the structure and content of the practice sessions during the 72-h post-match intervention to effectively manage the training load within this time frame (Silva et al., 2018). On the other hand, strength and conditioning coaches or sports scientists must include recovery monitoring as a daily routine, which allows the measurement of changes in fatigue/stress and recovery and prevention of overtraining or exposure to high levels of fatigue that can inhibit proper adaptation (Nédélec et al., 2012). Finally, every club must apply post-game recovery strategies (Nédélec et al., 2012), using the most modern recovery tools (Daab et al., 2020). In this context, the use of different recovery strategies offers significant positive effects in elite male soccer players (Altarriba-Bartes et al., 2020). However, young soccer players should not be considered young adults (Castagna et al., 2003). In fact, maturity differs among players of similar chronological age (Malina et al., 2004).

Children are able to resist fatigue better than adults during one or several repeated high-intensity exercise bouts (Ratel et al., 2006), and to recover from physical exertion faster than adults, especially, from high-intensity exercise (Falk and Dotan, 2006). These differences must be considered in the training and development of youth players. For instance, performance data derived from adults are not relevant to young soccer players, but the occurrence of acute (over the course of games) and residual (during fixture congestion) impairments in running performance in youth players may also be common (Palucci Vieira et al., 2019). Reductions in running performance over the course of a match do not appear to depend upon the peak height velocity (PHV) of the age group (Palucci Vieira et al., 2019), but congested match schedules have been shown to negatively affect the match running performance in young soccer players (Rowsell et al., 2011), specifically in post-PHV (Buchheit et al., 2011). During recovery period, significant relationships were also observed in young players between match running performance (total distance, high intensity, and sprinting distances) and physiological markers of muscle damage immediately after the match [creatine phosphokinase (CK), lactate dehydrogenase (LDH), and interleukin 6 (IL-6)] and 48 h (cortisol) post-competition (Aquino et al., 2016). However, in this population, the perceptual response throughout recovery is relatively unknown (Paul et al., 2019). Additional requirements with respect to adult players, such as study (which may inhibit the recovery process, possibly with additional mental fatigue imposed from lessons) or altered sleep quantity/quality ratio (Fullagar and Bartlett, 2016), may also influence the recovery pattern of young soccer players when they are working to obtain faster post exercise recovery abilities in pre-PHV athletes (Ratel et al., 2006).

Such as in older counterparts, prescription of post-match or post-training recovery strategies in young soccer players may be a key point to optimize soccer performance. However, the implementation of post-match recovery methods is only considered necessary in post-PHV players, and not in pre-PHV players (Buchheit et al., 2011; Rowsell et al., 2011). Considering the above information and that the effectiveness of recovery strategies may present interindividual variability (Tessitore et al., 2007), scientific evidence-based recovery methods and protocols used in adults are possibly not applicable to young soccer players. Hence, understanding the underlying mechanisms and plausible benefits of recovery strategies used in young players is also indispensable to help them achieve their maximum potential. Although recovery methods are now considered hot topics in the scientific literature, to the best of the authors' knowledge, the effects of different recovery strategies in young soccer players have not been deeply investigated. Therefore, the current systematic review primarily aimed to present a critical appraisal and summary of the original research articles that have evaluated the effectiveness of recovery strategies in young male soccer players and to provide sufficient knowledge regarding the effectiveness of the recovery methods and strategies to both coaches and players.

## Methods

### Information Sources

This article is a systematic review focusing on the recovery methods in young soccer players. The review was conducted following the Preferred Reporting Items for Systematic Review and Meta Analyses guidelines (Liberati et al., 2009). A structured search was conducted in WOS, PubMed, Cochrane Library, Evidence Database (PEDro), Evidence Based Medicine (EBM) Search review, EMBASE, and Scopus. The research ended on November 31, 2020. Search terms included a mix of medical subject headings (MeSH) and free-text words for key concepts related to recovery, young, soccer, and players. The following search equation was used to find the relevant articles: [“recovery”(MeSH Terms) OR “recover”(All Fields)] AND {[“young”(MeSH Terms) OR “youth”(All Fields)] OR “men”(All Fields) OR “male”(All Fields)]} AND {[“soccer”(MeSH Terms) OR “football”(All Fields)] OR [“sports”(MeSH Terms) OR “sports”(All Fields) OR “sport”(All Fields)]}. The search for published studies was independently conducted by two authors (JC-G and DM-J).

### Study Inclusion and Exclusion Criteria

The PICOS model was used to determine the inclusion criteria (O'Connor et al., 2008): P (Population): “Young soccer players,” I (Intervention): “Recovery methods,” C (Comparators): “identical conditions for experimental trials,” O (Outcome): “physical and/or neuromuscular performance measurements, physiological responses, and perceptual measures,” and S (study design): “controlled and randomized design.”

As a result, studies included in this systematic review had to meet the following inclusion criteria: (I) the study population comprised young male soccer players aged younger than 18 years; (II) participants used recovery methods; (III) the effects of the methods were compared with a control condition or between methods; (IV) articles examined the effects of recovery methods on physical measurements, physiological responses, perceptual measures, or cognitive function; and (V) study designs were randomized.

The following exclusion criteria were applied to the experimental protocols of the investigation: (I) studies conducted using participants with a previous cardiovascular, metabolic, or musculoskeletal disorder, (II) articles regarding other team sports populations without included or duplicated articles, and (III) abstracts, non-peer-reviewed papers, and book chapters.

### Study Selection

Titles and abstracts of publications identified by the search strategy were screened for a subsequent full-text review and were cross-referenced to identify duplicates. All trials assessed for eligibility and classified as relevant were retrieved, and the full text was peer reviewed (JC-G and JM-A). Moreover, the reference section of all relevant articles was also examined using the snowball strategy (Gentles et al., 2015). Based on the information within the full articles, the inclusion and exclusion criteria were used to select the trials eligible for inclusion in this systematic review. Disagreements were resolved through discussions between two authors (DM-J and IR).

### Data Extraction

Once the inclusion/exclusion criteria were applied to each study, the following data were extracted: study source (author/authors and year of publication); population of the sample, indicating the number of participants; methods; characteristics of the intervention; and significant differences between the study groups.

### Quality Assessment and Risk of Bias

To carefully consider the potential limitations of the included studies to obtain reliable conclusions, following the Cochrane Collaboration Guidelines (Higgins and Green, 2011), two authors independently assessed the methodological quality and risk of bias (D.M-J and J.M-A), whereas disagreements were resolved by third-party evaluation (JC-G). In the Cochrane Risk of Bias tool, the following items were included and divided into different domains: (1) selection bias (items, random sequence generation, allocation and concealment), (2) performance bias (blinding of participants and personnel), (3) detection bias (blinding of outcome assessment), (4) attrition bias (incomplete outcome data), (5) reporting bias (selective reporting), and (6) other bias (other sources of bias). The assessment of the risk of bias was characterized as low risk (plausible bias unlikely to seriously alter the results), unclear risk (plausible bias that raises some doubts about the results), or high risk (plausible bias that seriously weakens confidence in the results).

## Results

**Figure 1:**
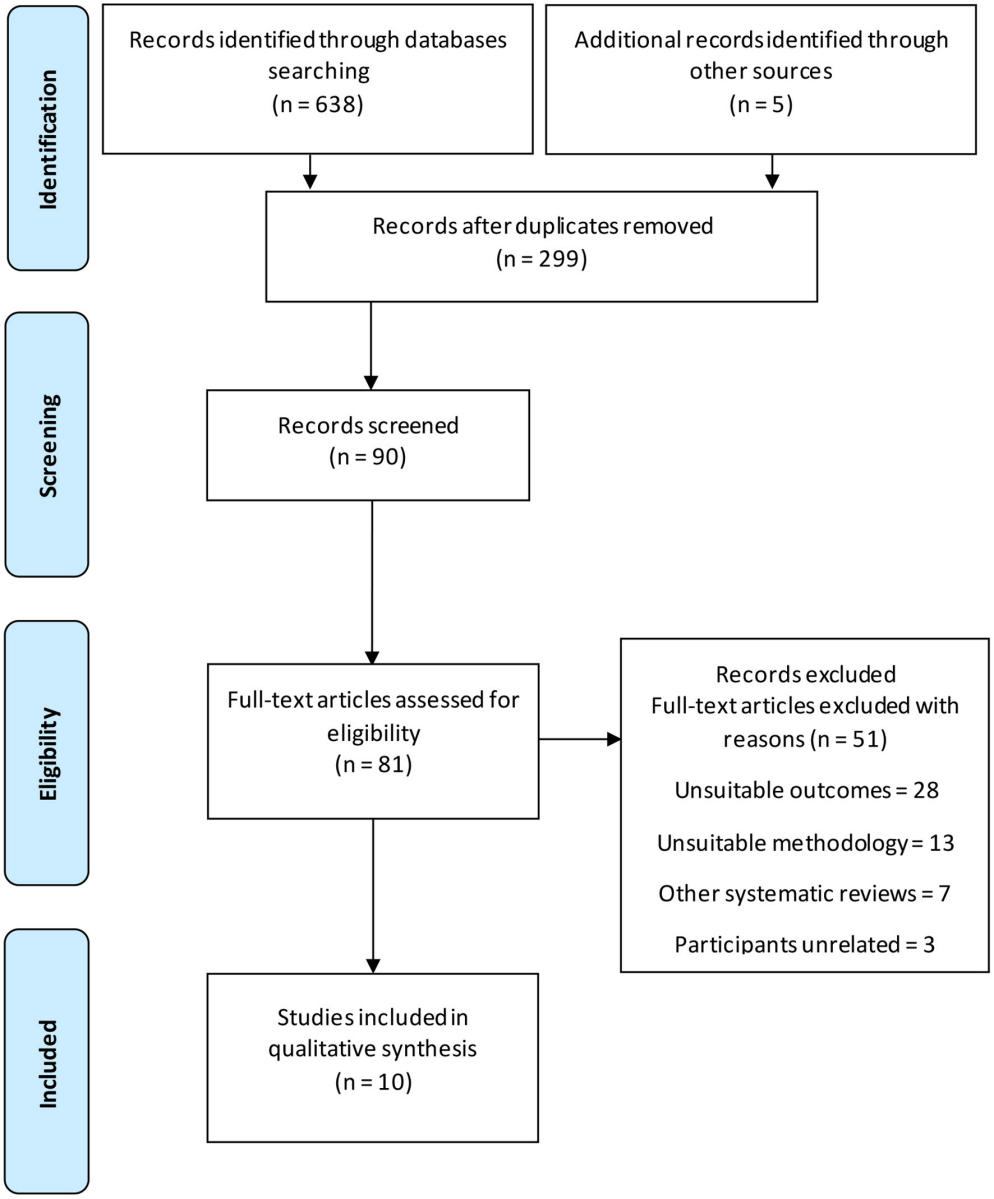
Flow diagram of the study selection.

The initial search of the scientific literature observed 638 soccer-related articles, but 299 articles were excluded given that they were unrelated to recovery in young soccer players (failed to meet the inclusion criteria) (Figure 1).

### Type of Recovery Strategy

A total of 10 studies were included (Table 1), which evaluated static stretching (SS) (Kinugasa and Kilding, 2009; Pooley et al., 2017, 2020); active recovery (AR) including jogging or submaximal exercise (Gharbi et al., 2017; Pooley et al., 2020; Trecroci et al., 2020); different water immersion strategies such as cold water immersion (CWI) (Rowsell et al., 2009, 2011; De Nardi et al., 2011; Pooley et al., 2020), contrast water therapy (CWT) (Kinugasa and Kilding, 2009; De Nardi et al., 2011), or thermoneutral immersion (TWI) (Rowsell et al., 2009, 2011); whole body vibration (WBV) (Marin et al., 2012); or a combined modality, which included CWI and AR (Kinugasa and Kilding, 2009) and a spa treatment, based on alternating thermotherapy and cryotherapy, sauna use, CWI, and Jacuzzi (Buchheit et al., 2011). To avoid misunderstandings, we have categorized the control group of some studies as passive recovery (PR, no recovery intervention), although we know this term can be used to classify recovery methods (Bompa, 1999).

**Table 1:** Recovery methods with positive effects in young soccer players.

**References**	**N/age/level**	**Method**	**Intervention protocol**	**Result**
Buchheit et al. (2011)	5 elite players 15.4 ± 0.4	Spa treatment PR	After 2-min shower (33–43°C, self-selected) the morning after match 1 (12–15 h post-match): - Spa treatment (combination of 3 techniques performed 3 times), which includes sauna (2 min, 85–90°C, seated), jacuzzi/hydromassage [2 min, hot water (36 ± 1.5°C), seated with water at the mid-sternal level], and CWI [2 min, cold water (12 ± 18°C), seated with water at the iliac crest/umbilicus level] - PR: no recovery intervention	Match running performance: - Total distance: Spa ↔ PR - Low-intensity running: Spa > PR - High-intensity running: Spa ↔ PR - Very high-intensity running: Spa ↔ PR - Sprinting distance: Spa > PR - Very high-intensity activities: Spa ↔ PR - Peak match speed: Spa > PR - Total number of sprints: Spa > PR - Number of repeated sprint sequences: Spa > PR
De Nardi et al. (2011)	18 regional league players 15.5 ± 1.0	CWT CWI PR	After each one of four training sessions during 4 consecutive days: - CWT: 2 × [2 min, cold water (15 ± 0.5°C) + 2 min, thermoneutral water (28 ± 0.5°C)] - CWI: 8 min, cold water (15 ± 0.5°C) to the iliac spine level - PR: 8-min rest	Neuromuscular performance: - CMJ: CWI ↔ CWT ↔ PR - RSA: CWI ↔ CWT ↔ PR Physiological parameters: - Uric acid: CWI ↔ CWT ↔ PR - Leucocytes: CWI ↔ CWT ↔ PR - Hemoglobin: CWI ↔ CWT ↔ PR - Reticulocytes: CWI ↔ CWT ↔ PR - CK: CWT and CWI < PRPsychological parameters: - RPE: CWI < CWT and PR
Gharbi et al. (2017)	10 amateur players 14.6 ± 0.8	AR PR	Two experimental sessions (including 2 shooting accuracy tests before and after a repeated dribbling sprint test) that differed only by recovery mode during the RDST: - AR: juggling exercise without using the athletes' arms or hands during the 20-s recovery - PR: during the 20-s recovery, players stood still	Soccer-specific performance: - Dribbling sprint time and total dribbling time: AR > PR - Fatigue index: AR > PR - Kicking accuracy: AR ↔ PR Physiological parameters: - Mean heart rate: AR > PR - (La): AR ↔ PR - Systolic/diastolic blood pressure: AR ↔ PR Psychological parameters: - RPE: AR > PR - Feeling scale: AR < PR
Kinugasa and Kilding (2009)	28 amateur players 14.3 ± 0.7	CWT CM SS	Three matches, each match randomly followed by 1 of 3 recovery modalities (2 single and 1 combined): - CWT: 3 × [1 min, cold water (12°C) to the level of the mesosternum + 2 min, hot shower (38°C)] - CM: 3 × [1 min, cold water (12°C) + 2-min active recovery 60–80 rpm/90–110 W) - SS: 7-min static stretching + 2 min legs raised above the heart level	Neuromuscular performance: - CMJA: CWT ↔ CM ↔ SS Physiological parameters: - HR: CM > CWT > SS at post-match, but not 24-h post-match - Tympanic temperature (°C): CWT and CM < SS post-match, but not 24-h post-match, CM > CWT at 24-h post-match Psychological parameters: - Perceived recovery (TQR): CM > CWT > SS at post-match, but not 24-h post-match - Thermal sensation: CWT < CM < SS at post-match, but not 24-h post-match - Heavy legs: CM and CWT < SS at post-match, CM < SS at 24-h post-match
Marin et al. (2012)	16 high-level players 17.1 ± 0.9	WBV Control cool down	RSA test before recovery strategy: - WBV: exercises performed with a vibration stimulus at high-H (50 Hz) or low-L (35 Hz), depending on the range of weights of the participants - Control cool down: same exercises without vibration stimulus	Neuromuscular performance: - CMJ: WBV > Control at 24-h post-RSA test - MVIC: WBV ↔ controlPsychological parameters: - DOMS: WBV < control
Pooley et al. (2017)	10 elite players 16 ± 1	SS PR	Minimum of three 80-min matches for each recovery intervention: - SS: two 15-s stretches to the gastrocnemius, hamstrings, quadriceps, glutes, hip flexors, adductors, and abductors - PR: 10-min passive seating	Neuromuscular performance: - CMJA: SS ↔ PRPhysiological parameters: - CK: SS ↔ PR - Oedema: SS ↔ PR Psychological parameters: - DOMS: SS ↔ PR
Pooley et al. (2020)	15 elite players 16 ± 1	CWI SS AR	Nine competitive soccer games, comprising three 80-min matches for each intervention: - CWI: 10 min, cold water (14 ± 0.8°C) to the iliac spine level - SS: two 15-s stretches to the gastrocnemius, hamstrings, quadriceps, glutes, hip flexors, adductors, and abductors - AR: 10-min low-intensity exercise on a cycle ergometer at 80–100 rpm/80 W	Neuromuscular performance: - CMJA: SS < AR and CWI Physiological parameters: - CK: SS > AR > CWI - Edema: SS ↔ AR ↔ CWI Psychological parameters: - DOMS: SS > AR and CWI
Rowsell et al. (2009)	20 high-level players 15.9 ± 0.6	CWI TWI	20 min after the end of each of the four matches for 4 consecutive days: - CWI: 5 × [1 min, cold water (10 ± 0.5°C) to the level of the mesosternum + 1-min seated rest on a chair at room 24°C] - TWT: 5 × [1 min, thermoneutral bath (34 ± 0.5°C) to the level of the mesosternum + 1-min seated rest on a chair at room 24°C)	Neuromuscular performance: - CMJ: CWI ↔ TWI - RSA: CWI ↔ TWI Physiological parameters: - Inflammatory markers (IL-1b, IL-6, IL-10): CWI ↔ TWI - EIMD markers (FABP, CK, LDH, Mb): CWI ↔ TWI Psychological parameters: - RPE: CWI ↔ TWI - General fatigue: CWI < TWI - DOMS: CWI < TWI
Rowsell et al. (2011)	20 high-level players 15.9 ± 0.6	CWI TWI	20 min after the end of each of the four matches during 4 consecutive days: - CWI: 5 × [1 min, cold water (10°C) to the level of the mesosternum + 1-min seated rest on a chair at room 24°C] - TWI: 5 × [1 min, thermoneutral bath (34°C) to the level of the mesosternum + 1-min seated rest on a chair at room 24°C]	Match running performance: - Total, distance: CWI > TWI - HIR distance: CWI ↔ TWI Physiological parameters: - HR (time in 80–90% HRmax): CWI > TWI only in matches 3 and 4 - HR (time in <80% HRmax): CWI < TWI only in matches 3 and 4 Psychological parameters: - General fatigue: CWI < TWI - DOMS: CWI < TWI
Trecroci et al. (2020)	9 subelite players 17.6 ± 0.5	AR Sport-specific training session (SST)	48 h post-match the players underwent the intervention (SST or AR) and 72 h post-match 30-m sprint, MVIC, and RSA were performed. - SST: warm-up, 20 min SSG, 15 min tactical drills, 10 min offensive/defensive set plays. - AR: training session at a lower exercise intensity lasting 30 min consisting of 15 min of circle drills, 5 min of dynamic stretching, 10 min of straight-line jogging (runs of 20 s interspersed by 40 s of walking recovery).	Neuromuscular performance: - 30-m sprint: SST ↔ AR - RSA: SST ↔ AR - MVIC (knee flexors): SST ↔ AR - MVIC (knee extensors): SST < AR

*↔ no statistically significant differences; > < significant effect compared with the following method*.

*AR, active recovery; CK, creatine phosphokinase; CM, combined modality; CMJ, countermovement jump; CWI, cold water immersion; CWT, contrast water therapy; DOMS, delayed-onset muscle soreness; FABP, Fatty acid-binding protein; HR, heart rate; IL-1b, interleukin 1b; IL-6, interleukin 6; IL-10, interleukin 10; LDH, lactate dehydrogenase; Mb, myoglobin; MVIC, maximal voluntary isometric contraction; PR, passive recovery; RPE, rating of perceived exertion; RSA, repeated sprint ability; SS, static stretching; TQR, total quality recovery scale; TWI, thermoneutral water immersion; WBV, whole body vibration; La, blood lactate concentration*.

### Type of Sample

These strategies were applied only in young male soccer players (total sample comprised 151 participants) and included elite level (Buchheit et al., 2011; Pooley et al., 2017, 2020), high level (Rowsell et al., 2009, 2011; Marin et al., 2012; Trecroci et al., 2020), or amateur/non-elite soccer players (Kinugasa and Kilding, 2009; De Nardi et al., 2011; Gharbi et al., 2017).

### Moment of Recovery Implementation

Studies included in this systematic review applied these methods between training sessions (De Nardi et al., 2011); after soccer matches (Pooley et al., 2017, 2020; Trecroci et al., 2020); during congested fixture schedules (Kinugasa and Kilding, 2009; Rowsell et al., 2009, 2011; Buchheit et al., 2011) between exercise bouts during training (Gharbi et al., 2017); or after a stimulus specific to soccer such as the repeated sprint ability (RSA) test (Marin et al., 2012).

### Type of Outcomes

Studies included in this systematic review measured a large range of variables (Table 1). Physical performance as a recovery marker was analyzed in seven studies (Kinugasa and Kilding, 2009; Rowsell et al., 2009; De Nardi et al., 2011; Marin et al., 2012; Pooley et al., 2017, 2020; Trecroci et al., 2020), physiological responses in seven studies (Kinugasa and Kilding, 2009; Rowsell et al., 2009, 2011; De Nardi et al., 2011; Gharbi et al., 2017; Pooley et al., 2017, 2020), and perceptual responses in eight studies (Kinugasa and Kilding, 2009; Rowsell et al., 2009, 2011; De Nardi et al., 2011; Marin et al., 2012; Gharbi et al., 2017; Pooley et al., 2017, 2020). However, match running performance was only evaluated in two studies (Buchheit et al., 2011; Rowsell et al., 2011), whereas technical skills during recovery period were measured once (Gharbi et al., 2017).

### Risk of Bias

**Figure 2:**
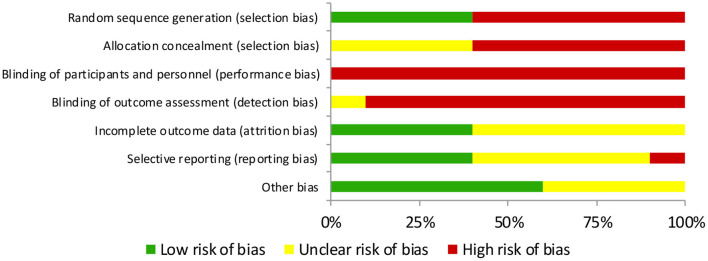
Risk of bias graph: review authors' judgments about each risk of bias item for each included study.

In relation to selection bias, random sequence generation and allocation concealment was characterized as high risk in the same six studies (Rowsell et al., 2009, 2011; Buchheit et al., 2011; Pooley et al., 2017, 2020; Trecroci et al., 2020), whereas random sequence generation was characterized as low risk, and allocation concealment was categorized as unclear in the rest of the included studies (Kinugasa and Kilding, 2009; De Nardi et al., 2011; Marin et al., 2012; Gharbi et al., 2017) (see Figure 2). Regarding performance bias, blinding of participants was categorized as high in all included studies (Kinugasa and Kilding, 2009; Rowsell et al., 2009, 2011; Buchheit et al., 2011; De Nardi et al., 2011; Marin et al., 2012; Gharbi et al., 2017; Pooley et al., 2017, 2020; Trecroci et al., 2020), whereas blinding of personnel was characterized as high in all studies (Kinugasa and Kilding, 2009; Rowsell et al., 2009, 2011; Buchheit et al., 2011; De Nardi et al., 2011; Marin et al., 2012; Gharbi et al., 2017; Pooley et al., 2020; Trecroci et al., 2020), except one, which was categorized as unclear (Pooley et al., 2017). The domain attrition bias, measured by incomplete outcome data, indicated that four studies were characterized as low risk (Buchheit et al., 2011; Marin et al., 2012; Gharbi et al., 2017; Trecroci et al., 2020), and six studies were considered to be of unclear risk (Kinugasa and Kilding, 2009; Rowsell et al., 2009, 2011; De Nardi et al., 2011; Pooley et al., 2017, 2020). In relation to reporting bias, which was evaluated through selective reporting, four studies were considered to be low risk (Kinugasa and Kilding, 2009; Buchheit et al., 2011; Gharbi et al., 2017; Trecroci et al., 2020), five were considered as unclear risk (Rowsell et al., 2009, 2011; Marin et al., 2012; Pooley et al., 2017, 2020), and one was considered as high risk (De Nardi et al., 2011). Finally, six studies were characterized as low risk of other bias (Kinugasa and Kilding, 2009; Buchheit et al., 2011; Gharbi et al., 2017; Pooley et al., 2017, 2020; Trecroci et al., 2020), and four studies had unclear risk (Rowsell et al., 2009, 2011; De Nardi et al., 2011; Marin et al., 2012) (see Table 2).

**Table 2:** Risk of bias summary: review of authors' judgments about each risk of bias item presented as percentages across all included studies.

**References**	**Sequence generation (selection bias)**	**Allocationconcealment (selection bias)**	**Blinding of participants (performance bias)**	**Blinding of personnel (performance bias)**	**Incompleteoutcome data (attrition bias)**	**Selective outcome reporting (reporting bias)**	**Other sources ofbias**
Buchheit et al. (2011)	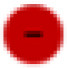	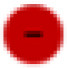	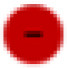	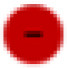	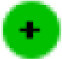	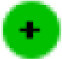	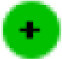
De Nardi et al. (2011)	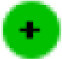	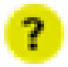	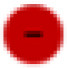	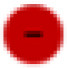	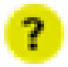	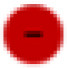	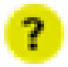
Gharbi et al. (2017)	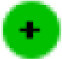	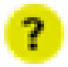	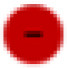	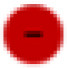	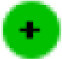	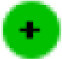	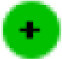
Kinugasa and Kilding (2009)	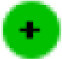	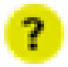	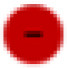	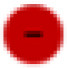	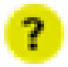	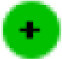	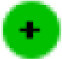
Marin et al. (2012)	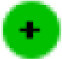	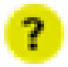	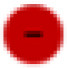	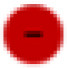	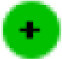	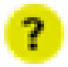	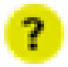
Pooley et al. (2017)	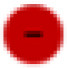	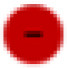	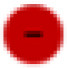	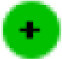	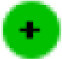	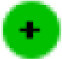	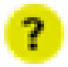
Pooley et al. (2020)	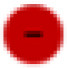	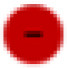	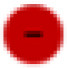	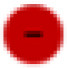	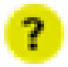	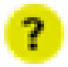	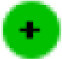
Rowsell et al. (2009)	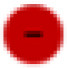	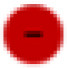	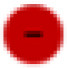	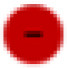	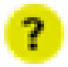	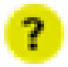	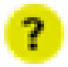
Rowsell et al. (2011)	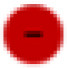	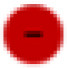	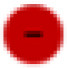	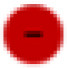	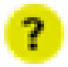	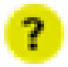	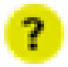
Trecroci et al. (2020)	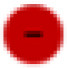	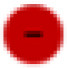	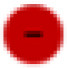	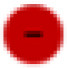	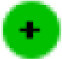	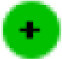	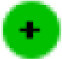
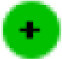 Indicates low risk of bias	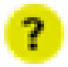 Indicates unclear risk of bias				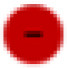 Indicates high risk of bias		

## Discussion

In order to provide a source of support for sport practitioners who work with young soccer players, this systematic review primarily aimed to examine the effects of different recovery methods on this population. The main conclusions were as follows: neuromuscular performance can be recovered using WVB but not with SS, and water immersion protocols may also be useful, but their positive effects are not significant; match running performance may also be maintained using water immersion protocols; EIMD and inflammatory responses could be positively affected when water immersion and AR are applied, although SS seems to be ineffective; perceptual responses are better with CWI and WVB, but contradictory results have been found when AR is applied, and SS had no positive impact.

Neuromuscular performance and physiological and perceptual responses have been considered as potentially useful for two purposes related to this topic: to detect changes in the time course response during recovery and to evaluate the efficacy and efficiency of recovery strategies. These variables have been measured in most of the studies included in this systematic review (Kinugasa and Kilding, 2009; Rowsell et al., 2009, 2011; De Nardi et al., 2011; Marin et al., 2012; Gharbi et al., 2017; Pooley et al., 2017, 2020). However, limited evidence exists on post-match recovery interventions to reduce decrements in match running performance during congested fixture schedules in young players (Buchheit et al., 2011; Rowsell et al., 2011), although this measure can be considered a more sensitive indicator of required physical ability than performance in simple field tests (Buchheit et al., 2011), even the most important (Palucci Vieira et al., 2019).

The evidence of the current literature investigating the effects of different recovery strategies is further discussed. To facilitate a practical application, this section is organized according to the effects obtained in young soccer players when recovery strategies are applied. Finally, recommendations are made for further investigation, and practical applications are exposed to help young soccer players achieve their maximum potential.

### Effects of Recovery Strategies on Neuromuscular Performance

One potential mechanism that may explain reductions in force production is the “popping sarcomere theory” associated to EIMD, which is caused following eccentric muscle contractions, where sarcomeres are overstretched beyond the point of filament overlap (Peake et al., 2017). This impaired muscle function negatively affects the quality of training sessions and therefore the subsequent adaptations, so enhancing recovery is vital to long-term training and performance (Marin et al., 2012). In this context, several interventions have been developed to reduce neuromuscular performance decrements of young players during recovery.

SS do not appear to be effective for neuromuscular performance recovery (Pooley et al., 2017) because SS exacerbates muscle damage by stretching further sarcomeres beyond the point of filament overlap (Pooley et al., 2020). Stretching of sarcomeres may open stretch-activated channels allowing calcium to enter the cytosol through these open channels of the sarcolemma, which may in turn stimulate the calpain enzymes to degrade contractile proteins (Peake et al., 2017), providing further reductions in force production. Moreover, both AR and CWI may improve neuromuscular performance in a greater extent than SS (Pooley et al., 2020). Despite this results, water immersion protocols (CWT, CWI, and TWI) may not significantly enhance CMJ performance recovery (Rowsell et al., 2009; De Nardi et al., 2011), although neuromuscular performance can be maintained better using CWT, CWI (De Nardi et al., 2011), or a combined recovery modality (CWI and AR) (Kinugasa and Kilding, 2009). In fact, combined modalities (WBV and SS) have been shown to have positive effects in lower-limb explosive force and CMJ recovery compared with single modalities (SS) (Marin et al., 2012).

The progressive decrease in RSA performance observed during successive matches or training sessions on consecutive days suggests that 24 h may be insufficient for the full restoration of physical performance (Rowsell et al., 2009; De Nardi et al., 2011), with 72 h an adequate time window to recover (Trecroci et al., 2020). Water immersion protocols (CWI, CWT, and TWI) have also failed to attenuate RSA decrements (decrease may be lower, but non-significant) in young soccer players during congested fixture schedules (Rowsell et al., 2009; De Nardi et al., 2011).

In an attempt to improve recovery process, coaches must also adjust the structure and content of the practice sessions during the 72-h post-match (Silva et al., 2018), and can be considered an interesting option to apply recovery methods. In this context, benefits of AR intervention applied 48 h after a single match may restore knee flexor muscle force production at a higher level compared with a soccer-specific training session, reducing injury risk at post-match period, but seems not to be considered a valid intervention to promote the recovery sprint performance, RSA, and muscle force production of knee extensors (Trecroci et al., 2020).

In summary, water immersion protocols may provide non-significant benefits to young soccer players' performances during recovery period, although it is unable to distinguish if any of the prescribed recovery modalities are better. WVB could be a valid option as well, but we recommend not to use SS if neuromuscular performance is the main goal of the recovery strategy prescription.

### Effects of Recovery Strategies on Match Running Performance

It has been reported that only post-PHV players experience match-induced fatigue within 48 h, as evidenced by a decreased match running performance during the following match, and therefore require recovery interventions, whereas the implementation of post-match recovery strategies in pre-PHV is questionable (Buchheit et al., 2011). To date, only water immersion strategies as a recovery option for match running performance maintenance have been evaluated. In this context, a spa sequence (sauna, Jacuzzi/hydromassage, and CWI) applied in post-PHV players the day after a match may have a beneficial impact on low-intensity running, sprinting distance, peak match speed, total number of sprints, and the number of repeated sprint sequences during the second match (Buchheit et al., 2011). However, it seems that CWI is better than TWI for recovery purposes in young soccer players because it can promote better maintenance of total match running distance in subsequent matches (Rowsell et al., 2011).

Physiological mechanisms remain unclear. On the one hand, performance could be influenced by psychological perception of well-being (Marcora and Bosio, 2007), which may predispose players to engage in more high-intensity exercise during the following match. Moreover, a plausible association may exist between the perception of fatigue and physical performance and the process in which athletes instinctively regulate their intensity of exercise based on the sensation of fatigue (Marcora et al., 2009). This may explain the positive effect of CWI on match running performance compared with TWI, as players exposed to CWI have reported less general fatigue and leg soreness compared with those players exposed to TWI (Rowsell et al., 2011). On the other hand, associated changes in intramuscular pressure and muscle blood flow, due to the repeated alternations of sauna and Jacuzzi (vasodilatation and improving feelings of well-being) and CWI (vasoconstriction, reduction of cell necrosis, reduction of edema, and neutrophil migration, which in turn reduce DOMS) (Prentice, 1999; Wilcock et al., 2006; Vaile et al., 2007), could also have facilitated the maintenance of match running performance.

As a result, it seems that it could not be a positive option to apply TWI alone (without CWI) when match running performance maintenance is the main objective of the recovery strategy with post-PHV players.

### Effects of Recovery Strategies on Soccer-Specific Performance

The precise influence of different recovery strategies on soccer-specific performance has not received consideration while studying different recovery modes in young soccer players. Only one study has been found (Gharbi et al., 2017), where the effects of AR (juggling exercise without using the athletes' arms or hands for 20 s) and PR between exercise bouts during training were compared. Results showed that a repeated dribbling sprint interspersed with PR resulted in a shorter total dribbling time and dribbling sprint time than those interspersed with AR, possibly due to a higher muscular reoxygenation, slower decline in oxyhemoglobin, slower phosphocreatine (PCr) depletion, and higher PCr resynthesis (Dupont et al., 2004). However, the best dribbling time and kicking accuracy were not affected by the recovery type. Consequently, young coaches are advised to utilize PR during training sessions requiring repeated high-intensity exercises if they want to maintain soccer-specific performance (Gharbi et al., 2017).

### Effects of Recovery Strategies on Physiological Responses

To attenuate EIMD and inflammatory response in young soccer players, different water immersion strategies, AR and SS have been investigated.

As expected, CK activity can increase after training sessions on consecutive days, but the increase is significantly lower when CWI or CWT is applied compared with PR, having no significant effect on uric acid, leukocytes, hemoglobin, and reticulocytes (De Nardi et al., 2011). Anyway, we cannot identify which water immersion method is more effective to attenuate EIMD biomarkers in young soccer players because no improvements on CK, LDH, myoglobin, and fatty acid-binding proteins have been reported during a soccer tournament using CWI or TWI (Rowsell et al., 2009). Both CWI and AR have been shown to attenuate CK response over a 48-h period compared with the conventional SS (Pooley et al., 2020). This may be due to the fact that exposure to CWI following exercise can potentially reduce lymphatic and capillary cell permeability through peripheral vasoconstriction induced by low temperatures and/or the effect of hydrostatic pressure (Wilcock et al., 2006), attenuating the efflux of CK from damaged muscle fibers. Moreover, and compared with SS, CWI protocol and AR may significantly assist in the removal of waste products and inflammatory cytokines (Barnett, 2006; Broatch et al., 2018). Consequently, SS seems not to have an effect on the repair and regeneration process after EIMD (Pooley et al., 2017), and the hypothesis that return to baseline may be achieved without an SS method must be accepted.

When water immersion is applied for recovery purposes, associated changes in intramuscular pressure and muscle blood flow can facilitate the reduction of inflammatory response to muscle damage, decreasing edema (Vaile et al., 2007). However, evidence does not support this effect in young soccer players because CWI or TWI is not effective in reducing the inflammatory response (IL-1b, IL-6, and IL-10) in the 24-h period after intense exercise (Rowsell et al., 2009). Similar to CWI, AR and SS are ineffective methods to reduce muscle edema in elite young players (Pooley et al., 2017, 2020).

Cardiovascular responses may also be different depending on the recovery strategy used. When AR is applied between repeated high-intensity exercises, the mean heart rate (HR) is significantly higher than that of PR, despite systolic and diastolic blood pressures at rest (Gharbi et al., 2017). Moreover, a combined modality (CWI + AR) applied between soccer matches may induce a higher HR than CWT and SS, with HR responses higher in CWT compared with SS (Kinugasa and Kilding, 2009). The underlying mechanisms can be related to the fact that blood flow increases to meet the demand of moderate-intensity exercise during recovery, total peripheral resistance remains significantly less, and the body increases metabolic rate with exposure to cold (Crisafulli et al., 2003; Shevchuk, 2007). Finally, regarding match running performance, it seems that CWI allows players to spend more time in the moderate and less time in the low HR zone than the players who used TWI as a recovery method (Rowsell et al., 2011).

In summary, EIMD biomarker responses are possibly attenuated when water immersion and AR strategies are applied for recovery purposes, but SS seems to be ineffective. Regarding inflammatory processes, more studies are required to identify which strategy shows positive effects because no strategy has been identified as the best option to modulate this response. Finally, it is important to consider that AR and water immersion strategies may modify HR response and therefore soccer performance.

### Effects of Recovery Strategies on Psychological Responses

Several methods have been applied to reduce DOMS and fatigue perception in young soccer players.

On the one hand, related to DOMS attenuation, it has been observed that WBV in combination with a traditional cool down protocol (SS) reduced the muscle pain induced by a soccer-specific effort in high-level junior players compared with SS (Marin et al., 2012). Moreover, there is limited evidence to suggest that SS alone may assist in the reduction of muscle soreness post-match in young soccer players (Pooley et al., 2017). This may be because their bodies are possibly accustomed to managing the repair of damaged muscle fibers and the removal of myoprotein (Brancaccio et al., 2007). In fact, when comparing the effects of recovery interventions on DOMS, AR and CWI demonstrated significantly greater effects than SS (Pooley et al., 2020), but differences between AR and CWI were not identified. Compared with TWI, young players who used CWI reported less leg soreness in a tournament (Rowsell et al., 2009, 2011). The underlying mechanisms may be related to the fact that CWI assists in reducing perceived soreness due to the reduced firing rate of the pain sensory receptors in the skin after cooling and due to vasoconstriction, which may reduce inflammation and the osmotic pressure of exudate, decreasing the pressure exerted on pain signaling nociceptors (Broatch et al., 2018).

On the other hand, young soccer players can report lower RPE values and fatigue perception when different water immersion treatments are applied. Players exposed to CWI have generally reported lower perception of fatigue compared with PR, CWT, and TWI after training sessions or during tournaments (Rowsell et al., 2009, 2011; De Nardi et al., 2011), although RPE responses may be similar between CWI and TWI (Rowsell et al., 2009). A combined modality (CWI + AR) may also elicit a moderately higher perceived recovery immediately after the recovery session than CWT and SS, with SS considered the worst method to improve perceived recovery (Kinugasa and Kilding, 2009). It is generally accepted that athletes perform better when they believe they received beneficial treatment (Beedie, 2007). Hence, the enhanced perceptual responses observed might have been observed because young players who used CWI believed that it improves recovery (placebo effect).

Considering the abovementioned results, DOMS may be reduced in young soccer players using WBV, CWI, and AR, but not with SS, whereas fatigue perception may be decreased mainly with CWI because AR or SS has no positive results.

### Future Research Lines

For future studies, and according to results published by Buchheit et al., the implementation of post-match recovery strategies in pre-PHV players may not be necessary (Buchheit et al., 2011). Hence, whether prescribing recovery modalities is better than not prescribing recovery modalities in pre-PHV soccer players should be confirmed. After answering this question, additional studies are required to evaluate the efficacy of the abovementioned recovery strategies in young soccer players, both in elite and non-elite population (including women), and between exercise bouts during training, after specific soccer exercises, after training sessions, after single soccer matches, and during congested fixture periods. Future studies should also investigate the effectiveness of a wider range of recovery strategies (such as nutritional strategies, neuromuscular electrical stimulation, compression garments, massage, foam rolling, hyperbaric oxygen therapy, infrared saunas, and sleep), which could include comparisons between a combined recovery modality and single recovery modalities. Physical performance, physiological measures, and psychological or perceptual states have been considered in previous publications, but limited evidence exists on the effect of recovery methods in match running performance during soccer matches and on its effect on technical skills (dribbling, shooting), even if they can be considered critical to performance in soccer.

### Practical Applications

Recovery strategies should be targeted against the major causes of fatigue, but evidence providing definitive conclusions that justify the prevalence of one or other recovery methods with young soccer players is lacking. Depending on the evidences of residual fatigue of each player and the main goal of the recovery strategy prescription, coaches and sports scientists must select those methods whose benefits have been previously reported, therefore individualizing recovery prescription. Considering that reason, this manuscript can help coaches and sports scientists identify and select the pertinent methods used to enhance recovery. However, the evidence-based recommendations for adults should be followed if other recovery methods that have not been included in this systematic review are going to be applied. Moreover, individually customizing the recovery modalities based on the preferences of the young soccer players is also considered beneficial.

## Conclusions

According to the included studies in this systematic review, several recovery strategies applied in young soccer players may have beneficial effects during recovery, both after trainings and matches or during congested fixture schedules. Neuromuscular performance can be recovered using WVB but not with SS, and water immersion protocols may also be useful, but their positive effects are not significant, and it is unable to distinguish the best water immersion method; match running performance maintenance may be achieved using water immersion protocols but no other recovery methods have been investigated; EIMD and inflammatory responses could be positively affected when water immersion and AR are applied, although SS seems to be ineffective; perceptual responses also seem to be better with CWI and WVB, but contradictory results have been found when AR is applied, and SS had no positive impact. Finally, it is important to consider that AR strategies may modify HR response and soccer-specific performance.

## Data Availability Statement

All datasets generated for this study are included in the article/supplementary material.

## Author Contributions

JC-G conceived and designed the investigation, analyzed and interpreted the data, drafted the paper, and approved the final version submitted for publication. JM-A and DM-J analyzed and interpreted the data. IR and ÁM-O critically reviewed the paper and approved the final version submitted for publication. SO, MD, and JS critically reviewed the paper. NT, JS, and MD approved the final version submitted for publication. All authors contributed to the article and approved the submitted version.

## Conflict of Interest

The authors declare that the research was conducted in the absence of any commercial or financial relationships that could be construed as a potential conflict of interest.
